# Context-dependent evaluation of prospective mates in a fish

**DOI:** 10.1007/s00265-015-1924-y

**Published:** 2015-04-25

**Authors:** Lisa Locatello, Federica Poli, Maria B. Rasotto

**Affiliations:** Department of Biology, University of Padova, Via U. Bassi 58/B, 35121 Padova, Italy

**Keywords:** Female preference, Comparative evaluation, Fish

## Abstract

Female choice is often assumed to be based on absolute preference, driven by a threshold value of mate attractiveness. However, increasing evidence suggests that females may instead perform a comparative evaluation of prospective mates, possibly incurring in violation of rational decision rules (e.g. independence from irrelevant alternative, IIA). A prototypical case is the ‘asymmetrically dominated decoy’ effect where the preference for a target option over a competitor is altered by the addition of an irrelevant alternative. Here, we test for this effect in the peacock blenny *Salaria pavo*. Females, in binary test (i.e. focal option dyad differing in body size and extension of a yellow spot), strongly preferred one of the options. The effect of decoys, asymmetrically dominating the focal options for either yellow spot extension or body size, varied according to the initially preferred trait and the decoy type. Indeed, the addition of a decoy caused a shift in preference only when the decoy exhibited the intermediate expression of the trait less preferred initially. By contrast, females did not modify their preference in the presence of the decoy for their preferred trait. Although females’ evaluation was context-dependent, the violation of IIA was clearly observed only with respect to the initially less preferred trait. This does not exclude that females are in any case using comparative decision rules. Indeed, when faced with three alternatives, two of which are proportionally closer to each other than to the third one, they might not be able to discriminate among them, perceiving stimulus absolute magnitude.

## Introduction

Theoretical and empirical studies have emphasized the adaptive significance of female mating preference, highlighting the quality of benefits gained by females in choosing a male in relation to the expression of his sexual traits (Andersson and Simmons 2006; Wagner 2011). In many species, females base their mating decisions on several male signals (Candolin 2003; Hebets and Papaj 2005); yet, how they combine the different male attributes to rank potential mates (Bateson and Healy 2005; Castellano et al. 2012) is not fully understood.

As mate choice plays a major role in determining their fitness, females are expected to have evolved the neural machinery necessary to identify the appropriate male, avoiding heterospecifics and favouring discrimination between conspecifics of different qualities (Grafen 1990). An implicit assumption, adopted in most models of mate choice, is that females base their choice on absolute decision rules, choosing the most attractive male that exceeds a minimal threshold (Jennions and Petrie 2007). However, increasing evidence of female plasticity in mate choice suggests that female decision mechanisms may be based on comparison of the alternatives available rather than on absolute stereotypic standards (Bateson and Healy 2005; Castellano et al. 2012). If female preference follows an absolute decision rule, their choice is predicted to obey the principle of independence from irrelevant alternative (IIA) (Bateson and Healy 2005; Castellano et al. 2012). This implies that the relative preference for two prospective mates is expected to remain unchanged, regardless of whether a third, inferior alternative is introduced (constant-ratio rule, Luce 1959), because this new option should take choices from the pre-existing options in proportion to their original shares. Moreover, the addition of an irrelevant third option should not determine an increase of the absolute preference for any of the options in the set (principle of regularity, Tversky and Simonson 1993). By contrast, a major implication of a comparative mate evaluation is that females can make irrational decisions, resulting as inconsistent across different choice contexts (Bateson and Healy 2005). Indications that females do not follow an absolute mechanism when ranking males’ attractiveness comes from different taxa, but clear evidence of irrational mate preferences is still lacking. In the Túngara frog, *Physalaemus pustulosus*, Kirkpatrick et al. (2006) showed that females do not use strict absolute mating preferences, but found no evidence of violations of rationality principles. Departure from absolute decision rules have been experimentally tested in the green swordtail, *Xiphophorus helleri* (Royle et al. 2008) and the fiddler crab, *Uca mjobergi* (Reaney 2009), too. In both species, female preference is context-dependent but does not conform to the expectations of the theoretical models. In the green swordtail, females show preference for the rare-male phenotype, a decision rule that may favour the maintenance of genetic variation, whereas in the fiddler crab, females seem to use a combination of absolute and comparative rules, assessing male traits differently when presented with prospective mates that vary in multiple traits.

Here, we test if females of the peacock blenny, *S. pavo*, evaluate mates relying on absolute values or operate through a comparison of alternatives across different contexts.

To this aim, we adopted an experimental approach focused to highlight if female preference is independent from irrelevant alternatives, a hallmark of rational decision-making (Bateson et al. 2003; Royle et al. 2008). We perform the classic test of ‘asymmetrically dominated decoy’ (Bateson et al. 2003; Royle et al. 2008; Reaney 2009), where the IIA is violated if the preference for a target option over a competitor is altered by the addition of a decoy option that is inferior to the target and the competitor on one attribute, but lies between them on a second. In particular, if choice depends on the quality of alternative rather than on absolute values, the asymmetrically dominated decoy is predicted to increase the preference for the option that dominates it on both attributes (Bateson et al. 2003; Bateson and Healy 2005). We estimated female preference for two male cues, the body size (S) and the area of the yellow spot on the head crest (Y), both assessed by females when evaluating prospective mates (Fagundes et al. 2007). In particular, we first evaluated female preference for a pair of options exhibiting opposite expression of S and Y (S: larger body size-smaller yellow area; Y: smaller body size-larger yellow area). Then, we tested the preference for the same pair of options in the presence of an asymmetrically dominated decoy for either of the following: (a) the yellow area (Dy), exhibiting the smallest body size but with an intermediate yellow spot area, or (b) the body size (Ds), exhibiting the smallest yellow spot but with an intermediate body size.

If female mating decision is based on a comparative evaluation mechanism rather than on absolute values, females should change their preference in the presence of an asymmetric decoy. In particular, if mate choice is context-dependent and the asymmetrically dominated decoy effect operates, we should record a violation of (a) constant-ratio rule, with an increase of the relative preference for the Y option when introducing Dy and a decrease of it in the presence of Ds; (b) regularity, with an increase of the absolute preference for Y option when Dy is added and for S option when adding Ds. By contrast, if mate choice is based on absolute decision rules, females should not change their preference among binary and trinary treatment groups, regardless of the number of choices or the type of decoys available.

## Methods

### Study species

The peacock blenny, *S. pavo*, is a medium-size fish (up to 14 cm total length) that inhabits the rocky shores of the Mediterranean Sea and adjacent Atlantic coast (Zander 1986). The mating system is promiscuous: males continuously receive eggs in their nest from different females and care for overlapping clutches (Patzner et al. 1986; Pizzolon 2010; Ros et al. 2010; Pizzolon et al. 2012), and females spawn with several males during the breeding season (Patzner et al. 1986). Nesting males are larger than females and exhibit a pronounced head crest that during the breeding season shows a yellow patch on both sides (Oliveira et al. 1999). Larger males may receive more eggs because they occupy better nests (Oliveira et al. 1999; Pizzolon 2010), but have been found to achieve higher mating success regardless of the size of their nest (Gonçalves et al. 2002b). Male body size is also positively related to the number of sperm produced (Pizzolon et al. 2012). Head crest size is informative of male’s fertility and parenting effort (Pizzolon et al. 2012). Moreover, it seems to convey information on different time scales since its general development reflects genetic and/or condition at the time of formation and growth, whilst the colour intensity of its yellow patch signals current health status (Locatello et al. 2012). Male mating success, in term of eggs received, appears to be influenced by specific male traits (Oliveira et al. 1999; Gonçalves et al. 2002a; Fagundes et al. 2007; Ros et al. 2010) as well as by his general attractiveness (Pizzolon et al. 2012). In choosing prospective males, females assess male body size (Fagundes et al. 2007), head crest size (these latter both positively related to male body size) (Gonçalves et al. 2002a; Gonçalves and Oliveira 2003) and coloured ornamentation (Locatello et al. 2012).

### Fish capture and maintenance

Peacock blenny females were captured during the breeding season in the Venetian Lagoon by scuba-divers using hand-held nets, and immediately transported to the lab where they were anaesthetized in a water solution of MS222 (Tricaine sulphate, Sandoz), measured (total length, TL, range 6.3–13.0 cm) and weighted (body weight range 8.2–16.5 g). From these measurements, we calculated a female body condition factor (*K*) = female weight/female total length^3^ (Fulton 1904). Females were then kept in groups of three/four individuals in 70-l tanks. All tanks were provided with sandy bottoms and artificial shelters. Females were transferred into individual experimental tanks for 2 days of acclimatization before trials. Water was renewed daily, temperature was maintained at 22 °C, and the light regime followed natural conditions. Fish were fed daily with fresh chopped *Mytilus* sp.

### Experimental design

**Fig. 1:**
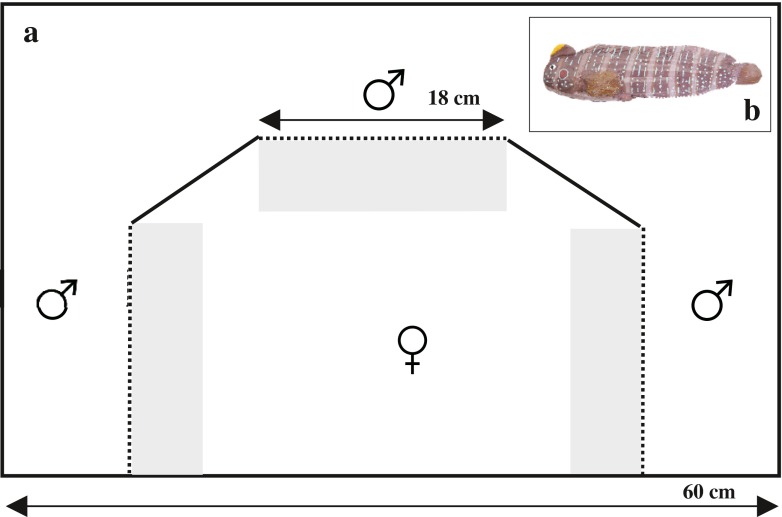
Mate choice arena (**a**) and male dummy (**b**). The rectangular tank (60 × 35 × 35 cm) was divided into two main compartments with a PVC partition; the central compartment hosted the tested female whilst each male dummy were placed laterally behind opaque removable partitions. *Dashed lines* represent removable opaque partitions and *solid lines* permanent opaque partitions. *Grey areas* define female preference zone (7 × 18 cm)

The mate preference experiments (*N* = 25) were performed in a trichotomous choice arena (Fig. 1a) (Royle et al. 2008) using male dummies (Fig. 1b), previously used successfully to investigate female preference in the peacock blenny (Locatello et al. 2012). A rectangular tank (60 × 35 × 35 cm) was divided into two main compartments with a PVC partition; the central compartment hosted the tested female whilst male dummies were placed laterally behind opaque removable partitions. Each dummy was attached alongside an artificial nest made of PVC tubes (15 cm long, 3 cm in diameter), to mimic natural breeding caves and to let the female perceive male body size and head crest characteristics. We designed 4 male dummies: (ì) focal S, the biggest in size (TL = 14 cm) and with a yellow spot covering 30 % of the crest area; (ìì) focal Y, with the largest yellow spot covering 90 % of the head crest area and TL of 10 cm; (ììì) decoy for size (Ds), intermediate in size (TL = 12 cm) and with a yellow spot smaller than the two focal options (10 % of the head crest area) and (ìv) decoy for yellow (Dy), intermediate in yellow spot area (70 % of the head crest area) and smaller in size than the two focals (TL = 8 cm). Head crest total area was designed proportionally to TL, according to data collected on natural males (Pizzolon 2010). Dummies were identically designed with respect to all other morphological traits, and their colour pattern was hand-painted. Each female was made to choose successively between the same S and Y options in 3 different contexts: (1) Y versus S, (2) Y versus S versus Dy and (3) Y versus S versus Ds. The practice of using the same dummies with all females could have suffered the problem of pseudoreplication (Kroodsma et al. 2001). However, as the dummies were hand-painted, small variation in colour patterns would have occurred if we would have used several dummies resembling the same stimulus type. In reusing the four dummies, we reduced the risk of unsuspected heterogeneity among them (Wiley 2003), thus avoiding to introduce a potential bias in female preference induced by the colour pattern of a particular dummy.

The ready to spawn female, exhibiting an enlarged belly and a red swollen genital papilla (Gonçalves and Oliveira 2003; Pizzolon et al. 2010), was placed in the central compartment of the tank 2 days before treatment to acclimatize. One hour before the experiment, three opaque partitions were inserted to visually separate the female, and the two focal dummies were randomly placed in two out of three compartments (Fig. 1). Opaque partitions were removed at the beginning of the experiment to allow visual interaction of females with dummies. Each trial lasted for 20 min, and successive trials were separated by 20 min of pause. At the end of the binary trial, the opaque partitions were replaced and the decoy was (Ds or Dy) added (in the first trinary trial) or changed (in the second trinary trial). In binary comparisons, male dummies occupied only two compartments; in trinary comparisons, all three compartments were occupied by dummies (Fig. 1). The binary choice came always first and was followed by the trinary trials with the decoy. The order of the two types of trinary trials (with Dy or with Ds) was randomized to control for the presentation order of the decoys. Moreover, dummies were moved randomly between compartments in the successive three trials to control for side bias. This allowed randomization also for the presentation of the empty compartment in binary trials. A female was defined as being in the preference zone (7 × 18 cm in front of dummies’ compartments. Fig. 1a) when her whole body was inside it (Pizzolon et al. 2010). We included in the analysis the females that, when entering the preference zone, positively responded to the dummies by showing the courtship behaviour typical of the species, i.e. showing rapid respiratory movements, pectoral fin fanning and displaying the ventral region (Patzner et al. 1986; Almada et al. 1995). Temperature and light were maintained constant throughout the experiments.

### Data analyses

The association time, i.e. the proportion of time spent in the preference zone of a dummy, a method commonly used to measure female preference in fish (Cummings and Mollaghan 2006; Royle et al. 2008; Walling et al. 2010), was used to evaluate female relative and absolute preferences. Preference indices were calculated following standard approaches used in comparative choice studies (see Shafir et al. 2002; Bateson et al. 2003; Royle et al. 2008). The relative proportions of time were used to test for violation of the constant-ratio rule and were calculated as the total amount of time a female spent in the preference zone of Y or S, divided by the total amount of time spent in the preference zones of both Y and S (i.e. time with S or Y/[time with S + time with Y], regardless whether a decoy was present or not). The absolute proportions of time spent with each focal dummy were used to test for violations of regularity of preference and were calculated as the total amount of times the option was chosen divided by the total amount of time spent in all preference zone (i.e. time spent with S or Y/[time with Y + time with S]’ for binary and S or Y/[S + Y + D]’ for trinary groups).

Proportions were arcsine square-root transformed, and normality of data was tested using the Kolmogorov–Smirnov test. The effect of the trials (binary, trinary with Ds, trinary with Dy) on female preference was tested using a linear mixed model (with restricted maximum likelihood estimation REML) in SPSS 21. We included female preference as the dependent variable, trial as a fixed factor and female TL and K as covariates. To account for repeated measures on individual females, female identity (ID) was included as a random factor with estimate of random intercepts for each subject. Tests of the fixed effects were followed by post hoc pairwise comparisons of estimated marginal means. *α* values were adjusted for multiple comparisons following Benjamini and Hochberg (1995). Variance components of the random effects were estimated on original, non-transformed data.

## Results

Although we did not observe any difference in the average time females spent with Y vs. S in the binary trials (paired *t* test: *t*_24_ = −0.74, *p* = 0.47; time with Y: mean ± SD = 281 ± 279 s; time with S mean ± SD = 225 ± 185 s), a strong bias in individual preference emerged. Twelve females spent 78.3 ± 18.9 % (mean ± SD) of the time with Y whilst 13 females spent 78.7 ± 18.9 % (mean ± SD) of the time with S.

When a strong initial bias in preference occurs, as shown in the green swordtail (Royle et al. 2008), the analyses of the whole data set could mask the effect of the decoy addition. In this case, the splitting of females into subgroups, based on their choice in binary comparisons, may highlight if the decoy presence drives the preference away from the preferred binary choice (Royle et al. 2008). Considering the pattern of preference recorded in binary tests, we analyzed our data splitting the females into two sub-groups according to the preference expressed in these tests. Peacock blenny females that in binary choice spent more than 50 % of their association time with the yellower dummy were assigned to the ‘yellow*’* female group, whereas those that spent more than 50 % of the association time with the larger dummy were assigned to the ‘size’ female group. *‘*Yellow*’* and *‘*size*’* females did not differ in TL (*t* test: *t*_23_ = −0.30, *p* = 0.77) and K (*t* test: *t*_23_ = 0.576, *p* = 0.57).

**Fig. 2:**
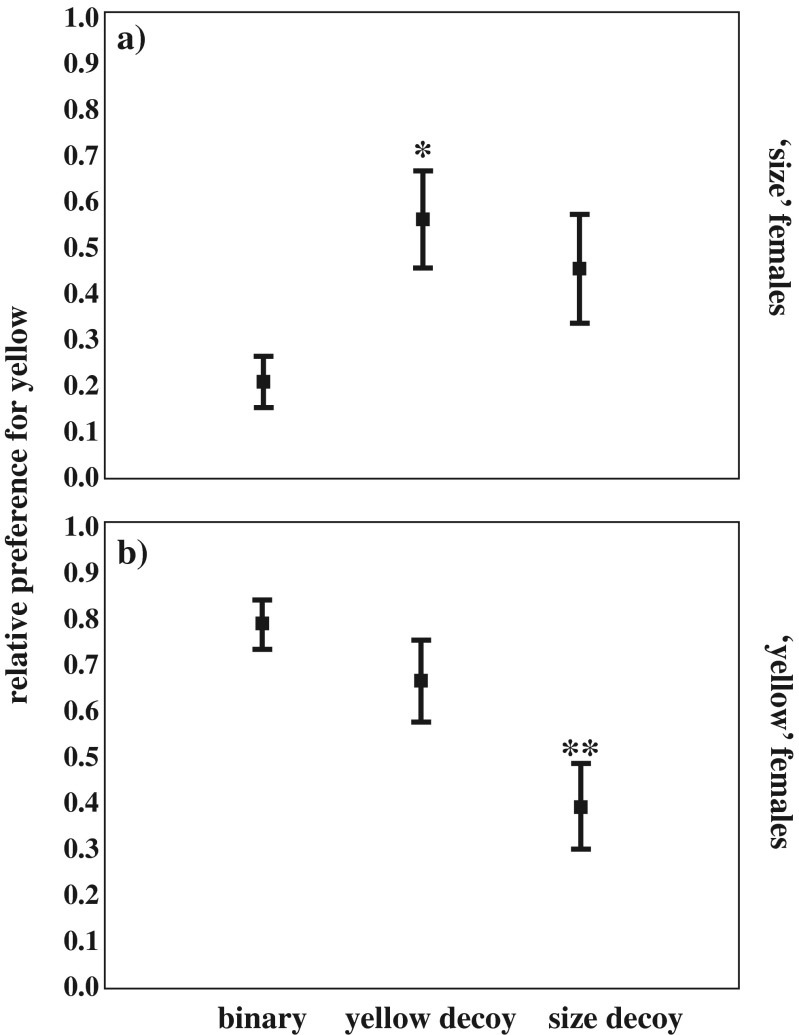
Relative proportion of female preference. Data are shown for **a** ‘size’ females that preferred S in the binary choice (*N* = 12) and **b** ‘yellow’ females that preferred Y in the binary choice (*N* = 13). Preference was evaluated in the three contexts: Y versus S, Y versus S versus Dy (yellow decoy) and Y versus S versus Ds (size decoy). Depicted are means ± SE. *p < 0.05; ** *p* < 0.01

The relative preference for Y over S was affected by the addition of the decoy in both the ‘yellow’ and the ‘size’ females (Table 1) (Fig. 2). Indeed, the latter increasingly preferred Y when Dy was present (*p* = 0.010, adjusted *α* = 0.025), whereas in the presence of Ds their shift in preference did not quite reach the statistical significance (*p* = 0.096, adjusted *α* = 0.05) (Fig. 2a). At the opposite, ‘yellow’ females’ preference for Y significantly decreased with the addition of Ds (*p* = 0.002, adjusted *α* = 0.025) whilst it did not change with the addition of Dy (*p* = 0.329, adjusted *α* = 0.05) (Fig. 2b).

**Table 1:** Results of the linear mixed models on relative and absolute preference of females that in binary choice spent more than 50 % of their association time with the yellower dummy (‘yellow’ females) and of females that in binary choice spent more than 50 % of the association time with the larger dummy (‘size’ females)

		Relative preference for Y over S			
		*F*	df	*p*	Variance component
‘Yellow’ females	Trial	5.731	2.34	0.007	
	Female TL	0.289	1.34	0.594	
	Female K	0.298	1.34	0.589	
	Female ID		−0.006 ± 0.014		
‘Size’ females	Trial	4.081	2.22	0.031	
	Female TL	0.011	1.9	0.920	
	Female K	0.046	1.9	0.835	
	Female ID				0.016 ± 0.026
		Absolute preference for Y			
		*F*	df	*p*	Variance component
‘Yellow’ females	Trial	14.983	2.34	<0.001	
	Female TL	0.838	1.34	0.366	
	Female K	0.030	1.34	0.862	
	Female ID				−0.017 ± 0.008
‘Size’ females	Trial	3.479	2.22	0.049	
	Female TL	0.397	1.9	0.544	
	Female K	0.002	1.9	0.968	
	Female ID				0.008 ± 0.017
		Absolute preference for S			
		*F*	df	*p*	Variance component
‘Yellow’ females	Trial	2.628	2.24	0.093	
	Female TL	0.009	1.10	0.925	
	Female K	0.051	1.10	0.825	
	Female ID				0.006 ± 0.011
‘Size’ females	Trial	12.950	2.22	<0.001	
	Female TL	0.112	1.9	0.745	
	Female K	0.006	1.9	0.940	
	Female ID				0.034 ± 0.026

**Fig. 3:**
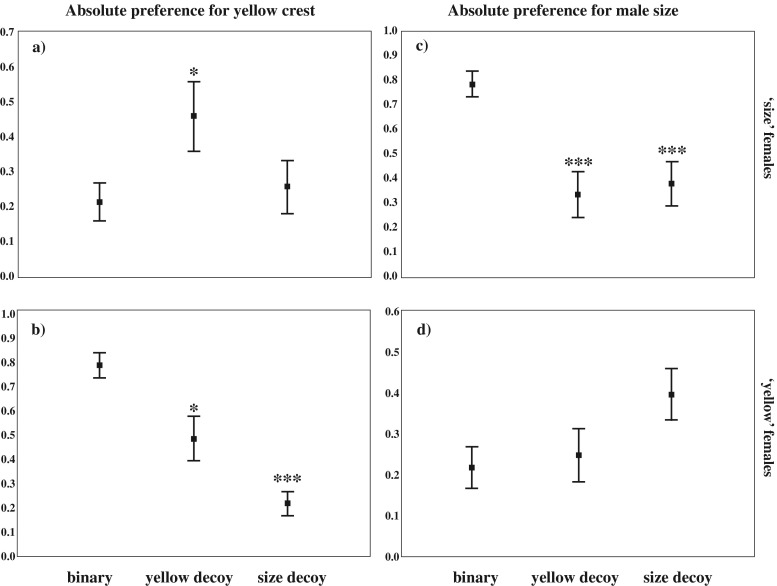
Absolute proportion of female preference. **a** and **c** Data are shown for ‘size’ females that preferred S in the binary choice (*N* = 12). **b** and **d** Data are shown for ‘yellow’ females that preferred Y in the binary choice (*N* = 13). Preference was evaluated in the three contexts: Y versus S, Y versus S versus Dy (yellow decoy) and Y versus S versus Ds (size decoy). Depicted are means ± SE. *p < 0.05; ** *p* < 0.01; ***p < 0.001

The decoys’ presence influenced also the mean absolute female preference. The absolute preference for Y was affected with the decoy addition in both the ‘yellow*’* and the ‘size*’* females (Table 1) (Fig. 3a, b). The absolute preference for S was significantly affected by the decoy addition in the ‘size*’* females (Table 1) whilst, in the ‘yellow*’* females, the change in absolute preference was not statistically significant (Table 1) (Fig. 3c, d). Indeed, the ‘size’ females, in the presence of Dy, increased their absolute preference for Y (*p* = 0.023, adjusted *α* = 0.025, Fig. 3a) and decreased that for S (*p* < 0.001, adjusted *α* = 0.025, Fig. 3c), whilst with Ds, the preference for Y remain unchanged (*p* = 0.721, adjusted *α* = 0.05, Fig. 3a) and that for S decreased (*p* < 0.001, adjusted *α* = 0.05, Fig. 3c). A similar pattern emerged from the tests with the ‘yellow’ females that in the presence of Ds decreased their absolute preference for Y (*p* < 0.001, adjusted *α* = 0.025, Fig. 3b) and increased their absolute preference for S, although slightly over the limit of statistical significance (*p* = 0.052, adjusted *α* = 0.025, Fig. 3d), whilst with Dy, the preference for S remained unchanged (*p* = 0.671, adjusted *α* = 0.05, Fig. 3d) and that for Y decreased (*p* = 0.006, adjusted *α* = 0.025 Fig. 3b). The absolute preference values, for both *‘*size*’* and *‘*yellow*’* females, were influenced by the relevant amount of time spent by females in inspecting the decoy that showed an intermediate expression of the trait preferred in binary test (size females: 38 % of time with focal S and 35 % with Ds; yellow females: 48 % of time with focal Y and 27 % with Dy).

In all the LMM, the variance components for the random effects (female ID) did not significantly differ from zero (−2 log likelihood ratio test with df = 1).

## Discussion

Overall, our results revealed that, in the peacock blenny, female preference is context-dependent. Females vary in the initial attention they pay to larger or more colourful males and continued to show differences in response to different decoys. Indeed, the addition of a third alternative caused an increase of both absolute and relative preferences only when the asymmetrical decoy exhibits the intermediate expression of the trait initially less preferred. In this case, violations of both constant-ratio rule and regularity occurred, in the direction expected from models of comparative evaluation (Bateson and Healy 2005). By contrast, in all females, the relative preference for the original option remained unchanged and the absolute one decreased in the presence of the more similar decoy, as females devote a considerable amount of time valuating it. These results could be indicative of structural constraints in female expression of mate preference or in their perception abilities. The initial preference that females expressed for one option was always very strong, being on average over 78 % of the total time. It may be possible that this preference has reached a ceiling and could not be easily increased. On the other hand, the long time females spent with the decoy exhibiting the intermediate expression of the trait preferred in the binary test suggests that females may be limited in discriminating the trait magnitude. Indeed, peacock blenny female discrimination between two stimuli might depend on the ratio of the two stimulus quantities rather than on their absolute difference, as predicted by Weber’s Law and recently demonstrated in a variety of taxa (Akre et al. 2011; Akre and Johnsen 2014). In such a scenario, the minimum difference between two quantities for discrimination is greater when stimulus quantity is high compared to when it is low (Akre and Johnsen 2014). In our trinary tests, the trait expression of both the decoy and one focal were high, with the decoy for the yellow area exhibiting a crest spot 22 % smaller than that of the focal for this trait and the decoy for body size being 14 % smaller than the larger focal. Thus, we cannot exclude that Weber’s Law might have influenced the preference when the decoy was more similar to the preferred focal trait than when the decoy was more similar to the trait that did not trigger the overall preference of the female. In this respect, it is interesting to notice that ‘size*’* females, in the presence of two expressions, differing in absolute values but proportionally similar in their preferred trait, spent an almost equal amount of time with the decoy and the focal option.

The few studies on irrational mating decisions (Kirkpatrick et al. 2006; Reaney 2009; Royle et al. 2008; present one) show that female choice may be plastic and context-dependent, based on preference rules more complex than previously assumed. On a theoretical level, the comparative evaluation of prospective mates is expected to have been favoured in species where simultaneous encounters of males are common (Castellano et al. 2012). With this respect, in the green swordtail (Royle et al. 2008), the fiddler crab (Reaney 2009) and the peacock blenny, i.e. the species where females’ preference is context-dependent, females have the opportunity to simultaneously face a number of potential mates that differ in more than one trait. Indeed, the green swordtail occurs at a high population density in its natural habitats (Simmons et al. 2008); in the fiddler crab, several males cluster around a single female and display their enlarged claw (Reaney 2009), and in the peacock blenny population used in the present study, males nest at a density of up to six individuals/square meter (Pizzolon 2010).

Choosing mates on the basis of absolute, rational rules has been considered by traditional models as the behaviour that maximizes benefits (Jennions and Petrie 2007). However, the patterns emerging from the studies on the decision-making mechanisms underlying female mating preferences show that choice may be context-dependent (Kirkpatrick et al. 2006; Reaney 2009; Royle et al. 2008; present one), deviating from the assumptions of rational choice theory (Luce 1959; Tversky and Simonson 1993). Departures from rational behaviour in foraging choices have been recently proposed to be adaptive, representing optimal strategies to exploit food options that vary in space and time (McNamara et al. 2014; Fawcett et al. 2014). Similarly, to understand female flexibility in mating preferences, future theoretical and experimental studies should focus on the functional role of decision-making mechanisms, evaluating if absolute or comparative rules are used and in which scenarios they represent adaptive responses to environmental problems.
